# Antibacterial Activity of Estragole From *Ocimum tenuiflorum*, and *Tagetes lucida* and Synergistic Effect With Meropenem and Tobramycin Against Multidrug‐ and Extensively Drug‐Resistant (MDR‐XDR) Gram‐Negative Bacteria

**DOI:** 10.1002/mbo3.70253

**Published:** 2026-02-23

**Authors:** María Araque, Luis B. Rojas

**Affiliations:** ^1^ Laboratory of Molecular Microbiology, Faculty of Pharmacy and Bioanalysis University of Los Andes Mérida Venezuela; ^2^ Research Institute, Faculty of Pharmacy and Bioanalysis University of Los Andes Mérida Venezuela

**Keywords:** antimicrobial, estragole, meropenem, multirresistant bacteria, synergy, tobramycin

## Abstract

Estragole, a prime compound present in the essential oils (EOs) of *Ocimum tenuiflorum* and *Tagetes lucida*, shows significant antimicrobial activity against WHO priority bacterial pathogens. This study evaluated the bactericidal effects of estragole, both in its chemically pure form and when isolated from the EOs of these plants, when used alone and in combination with the antibiotics meropenem and tobramycin against multidrug‐resistant (MDR) and extensively drug‐resistant (XDR) Gram‐negative bacteria. Antibacterial activity was evaluated using the minimum inhibitory concentration (MIC) and minimum bactericidal concentration (MBC) methods. The interaction of estragole with the selected antibiotics was analyzed using the checkerboard method, where the fractional inhibitory concentration index (FICI) was calculated. Results showed that estragole has inherent bactericidal properties, with MICs of no more than 256 μg/mL, indicating significant activity regardless of the bacteria's resistance profile. Combinations with meropenem and tobramycin exhibited pronounced synergy, achieving reductions in the MIC of up to 16‐fold in over 88% of the bacteria tested. An additive or partially synergistic effect was evident in only around 11% of cases. These results highlight the potential of estragole as a therapeutic adjuvant capable of enhancing the efficacy of conventional antibiotics, reducing the required doses and the associated side effects and toxicities. Furthermore, this study emphasizes the beneficial potential of phytochemical‐antibiotic combinations as an innovative strategy to address the growing threat posed by antimicrobial resistance.

## Graphical abstract

This study reveals estragole's bactericidal activity against MDR/XDR Gram‐negative pathogens. Synergy with meropenem and tobramycin was observed in > 88% of strains, reducing antibiotic MICs up to 16‐fold. Estragole represents a promising therapeutic adjuvant to enhance antibiotic efficacy and combat antimicrobial resistance.

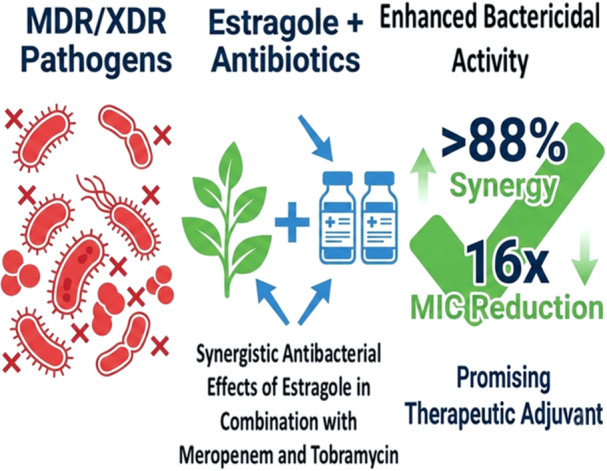

## 1 Introduction

Antimicrobial resistance (AMR) has emerged as a critical global health and socioeconomic challenge that needs immediate action (WHO 2022). Antibiotic‐resistant bacterial infections claimed approximately 1.27 million deaths around the world in 2019 (Murray et al. 2022). However, the WHO estimated that by 2050, infections caused by multidrug‐resistant bacteria could cause 10 million deaths per year, surpassing cancer as the leading cause of death (Salam et al. 2023). In 2024, the WHO updated the list of bacteria that should be considered a priority due to the limited treatment alternatives available and their impact on global health. At the top of this list are critical priority bacteria, including carbapenem‐resistant *Acinetobacter baumannii*, carbapenem‐resistant *Enterobacter*, and third‐generation cephalosporin‐resistant Enterobacterales (WHO 2024). Infections caused by these pathogens are difficult to prevent, highly transmissible, and have a high mortality burden, making them a major public health threat (Mancuso et al. 2021).

The growing concern about antibiotic resistance has prompted research on new therapeutic alternatives, as well as optimizing the effectiveness of existing treatments (WHO 2024; Salam et al. 2023). In this context, higher plants and their derived products can provide an enormous variety of structurally diverse complex compounds with a broad spectrum of action. Indeed, a significant proportion of modern pharmaceuticals contains one or more substances derived from plants, or are chemically synthesized from them (Swamy et al. 2016). In this regard, it is noteworthy to mention the substantial investigations conducted on essential oils (EOs), which are complex biochemical mixtures extracted from aromatic plants. These studies have shown that EOs exhibit a broad spectrum of antimicrobial activity against human pathogens, as well as other biological functions such as antioxidant or anti‐inflammatory activity (Brandes et al. 2024). The antimicrobial potential of EOs has been recognized for a long time, and comprehensive studies on the components responsible for this activity and their mechanisms of action are now available (Boren et al. 2020; Rajčević et al. 2022). EOs may not only be effective on their own as antimicrobial but also have the potential to act synergistically with conventional antibiotics (Suganya et al. 2022). Recently, the combination of antibiotics with EOs from higher‐plant by‐products has become a feasible strategy and an innovative approach for the treatment of infections caused by resistant pathogens (Diniz do Nascimento et al. 2020; Kashyap 2024). The advantages of this combination lie in its ability to boost the antibacterial activity of antibiotics. By doing so, it can reduce the required doses, which may decrease toxicity and incidence of side effects. This reduction, in turn, lessens the selective pressure that contributes to the development of resistant strains (Cheesman et al. 2017).

Estragole, also known as *p*‐allylanisole, methylchavicol or 4‐allylanisole, is a phenylpropene compound that is widely used in the food industry and in the preparation of fragrances (Azizah et al. 2023). It is an important chemical constituent of EOs of many aromatic plants, including species of the genus *Ocimum* and *Tagetes*. Experimental studies have shown that estragole is a bioactive molecule with antioxidant, antimicrobial, and insecticidal effects (Shirazi et al. 2014; Mahendra et al. 2023). This phytochemical is known to possess significant antimicrobial properties with the capacity to inhibit the growth of various bacterial strains, including those that show resistance to multiple antibiotics. Its mode of action involves the disruption of the bacterial cell membrane integrity, which results in cellular lysis and death (Diniz do Nascimento et al. 2020; Bordón et al. 2025). Additionally, it interferes with the metabolic pathways of microorganisms, increasing its potential as a therapeutic agent (Kashyap 2024). Moreover, the synergistic impact of estragole when used in combination with conventional antibiotics suggests an important adjuvant effect, enhancing the therapeutic efficacy of antimicrobial agents (Alam et al. 2022; SeyedAlinaghi et al. 2025). In this context, recent studies have revealed that this compound, when used in conjunction with antimicrobial agents, can significantly reduce the minimum inhibitory concentration (MIC) of antibiotics such as ampicillin, cefriaxone, gentamicin, and ciprofloxacin, increasing efficacy against resistant strains of *Enterococcus* (Medeirosa et al. 2023). In an experimental model using *Drosophila melanogaster*, it was demonstrated that 4‐allylanisole inhibited bacterial resistance mechanisms mediated by the production of beta‐lactamase and the efflux protein QacA/B, thus reducing penicillin MICs and improving antimicrobial sensibility in *Staphylococcus aureus* strains (Pereira Lopes et al. 2022). Another study concluded that the estragole‐β‐cyclodextrin complex associated with gentamicin, an aminoglycoside widely used in veterinary and human medicine, exhibited relevant antibacterial activity and a significant synergistic effect with gentamicin in reducing bacterial load in *S. aureus* and *Escherichia coli* infections in an experimental model with zebrafish (Batista et al. 2023). Moreover, it is important to note that there is currently no data available on the potential for synergies between phytocompounds and antibiotics to generate resistance, nor on their adverse environmental impact (Gan et al. 2023).

The genus *Ocimum* consists of over 150 species and has a wide geographical distribution. It is native to South Asia and belongs to the Lamiaceae family (Yamani et al. 2016; Azizah et al. 2023). In Venezuela, there is an abundance of species: *O. basilicum* Linn var *basilicum*, *O. basilicum* var *purpurescens*, *O. gratissimum* L, and *O. tenuiflorum* L. Acosta et al. (2003) conducted a chemical characterization of the EOs derived from multiple species of *Ocimum*, ascertaining that the predominant component was identified as 4‐allylanisole (> 95%), exhibiting an inhibitory effect on various multidrug‐resistant (MDR) bacteria of nosocomial origin.

The genus *Tagetes* belongs to the Asteraceae family and includes about 56 species distributed in the Americas (Armas et al. 2012). *T. lucida* Cavanilles is known for its ceremonial use and traditional medicinal applications. Previous research has shown that this species possesses antibacterial properties against Enterobacterales as well as fungicidal and cytotoxic effects against dermatophytes and various species of *Aspergillus*, *Penicillium*, and *Fusarium* (Joshi and Barbalho. 2022). Studies of the chemical composition of the EO of *T. lucida* have identified estragole as the major component (> 90%) (Salehi et al. 2018; Bordón et al. 2025).

Research on the interactions between phytocompounds and antibiotics has revealed that these combinations can enhance the efficacy of treatments, suggesting a promising avenue for the development of new therapeutic strategies (Silva et al. 2019; Boren et al. 2020; Kashyap 2024). However, studies on the use of estragole to increase the effect of antibiotics to treat infectious diseases are scarce. Therefore, the present study evaluated the antibacterial activity of estragole obtained from *O. tenuiflorum* and *T. lucida*, as well as its synergistic effect with antibiotics such as meropenem and tobramycin, against MDR Gram‐negative bacteria of high clinical priority. This study aims to develop new therapeutic strategies to combat the growing threat of bacterial resistance.

## 2 Materials and Methods

### 2.1 Phytocompound and Antibiotics

EOs of *O. tenuiflorum* (EOT) and *T. lucida* (ETL) were provided by the Research Institute of the Faculty of Pharmacy and Bioanalysis of the University of Los Andes, Mérida, Venezuela. These EOs were previously chemically characterized by Gas Chromatography‐Mass Spectrometry (GC‐MS) techniques and were selected for this study due to their high estragole content (> 95%) (Table 1). Chemically pure estragole purchased from Sigma‐Aldrich (USA) was used as a quality control (EQC) phytocompound. A stock solution was made for each of the EOs and the commercial product using 400 mg/mL (w/v) in dimethylsulfoxide (DMSO; Thermo Fisher Scientific, Inc. USA) and phosphate buffer saline (PBS; Thermo Fisher Scientific, Inc. USA). All stock solutions were placed in hermetically sealed bottles, protected from light and kept at 4°C. Meropenem (MER) and tobramycin (TOB) laboratory standards were purchased from Sigma‐Aldrich (USA).

**Table 1:** Chemical composition of the essential oils of *O. tenuiflorum* and *T. lucida*.

		Peak area (%)	
Compounds^a^	KI	*O. tenuiflorum*	*T. lucida*
4‐allylanisole (Estragole)	1195	**97.6**	
4‐allylanisole (Estragole)	1207	—	**95.5**
Myrcene	988	—	0.4
Trans‐β‐caryophyllene	1435	—	0.4
Trans‐β‐farnesene	1471	—	2.5
Germacrene D	1499	—	0.7
	**Total (%)**	97.6	99.5

a Chemical composition was determined by comparison of the mass spectrum of each component with the Wiley GC/MS library database and from retention index (RI) data. Abbreviation: KI, Kóvats index.

### 2.2 Bacterial Strains

A total of 12 Gram‐negative bacterial strains were evaluated in this study. Two of these were part of the American Type Culture Collection (ATCC) and corresponded to the category of bacteria that exhibited sensibility to antimicrobials (*E. coli* ATCC 25922 and *P. aeruginosa* ATCC 27853) and represented quality control of the susceptibility antimicrobial tests. The remaining 10 strains were grouped according to their resistance profile into two categories: MDR bacteria (*E. coli* LMM‐19B2, *Enterobacter cloacae* LMM‐U337, *K. pneumoniae* LMM‐2009, *K. pneumoniae* LMM‐029, and *Salmonella* Heidelberg LMM‐179) and extremely resistant bacteria (*E. coli* LMM‐A717, *K. pneumoniae* LMM‐U2023, *A. baumannii* LMM‐U496, *P. aeruginosa* LMM‐022, and *P. alcaligenes* LMM‐14249/2). All strains exhibiting resistance profiles are of clinical origin and have been previously characterized genetically and microbiologically in previous studies (Table 2). These strains are part of the collection of the Molecular Microbiology Laboratory of the Faculty of Pharmacy and Bioanalysis of the University of Los Andes, Mérida, Venezuela.

**Table 2:** Characteristics of bacterial strains used in this study.

N° Strain	Bacteria	Susceptibility profile	Reference
Susceptible (S)			
ATCC 25922	*Escherichia coli*	Susceptible	ATCC
ATCC 27853	*Pseudomonas aeruginosa*	Susceptible	ATCC
Multi‐drug resistant (MDR)			
LMM‐19B2	*Escherichia coli*	ESBL (CTX‐M‐15); CIP; TET	Araque and Labrador (2018)
LMM‐U337	*Enterobacter cloacae*	ESBL (CTX‐M‐1); CIP; GEN	Salazar and Araque (2023)
LMM‐2009	*Klebsiella pneumoniae*	ESBL (SHV‐2); CIP; TET; CLF	Barreto et al. (2009)
LMM‐029	*Klebsiella penumoniae*	ESBL (CTXM‐1 and CTXM‐2); CIP; GEN; FOS	Millán et al. (2013)
LMM‐179	*Salmonella* Heidelberg	ESBL (CTX‐M‐2); CIP; GEN; TET; SXT	González and Araque (2019)
Extensively drug‐resistant (XDR)			
LMM‐A717	*Escherichia coli*	ESBL (CTX‐M‐2); KPC (Type‐2); CIP; GEN; NIT; FOS	Millán et al. (2020)
LMM‐U2023	*Klebsiella pneumoniae*	ESBL (CTX‐M‐5); MBL (VIM‐1); CIP; GEN; SXT; FOS	Millán et al. (2013)
LMM‐U496	*Acinetobacter baumannii*	MBL(VIM‐2); CIP; GEN; AMK; FOS	Quijada‐Martínez et al. (2017)
LMM‐022	*Pseudomonas aeruginosa*	MBL (VIM‐1); CIP; AMK; GEN; TOB; FOS	El Hindawi et al. (2023)
LMM‐14249/2	*Pseudomonas alcaligenes*	AmpC; ESBL (SHV‐5); CIP; SXT; TET; FOS	Flores‐Carrero et al. (2016)

Abbreviations: AMK, amikacin; AmpC, beta‐lactamase (like cephalosporinases); ATCC, American Type Culture Collection; CIP, ciprofloxacin; CLF, chloramphenicol; CTX‐M, extended‐spectrum beta‐lactamase type cefotaximase; ESBL, extended spectrum beta‐lactamase; FOS, fosfomycin; GEN, gentamicin; KPC, *Klebsiella pneumoniae* carbapenemase; LMM, Laboratory of Microbiology Molecular; MBL, metalo‐beta‐lactamase; NIT, nitrofurantoin; SHV, extended spectrum beta‐lactamase type Sulphydryl Variable; SXT, trimethoprim‐sulfamethoxazole; TET, Tetracycline; TOB, Tobramycin; VIM, verona integron‐encoded metallo‐beta‐lactamase.

### 2.3 Determination of Minimum Inhibitory Concentration (MIC) and Minimum Bactericidal Concentration (MBC)

The MIC of EOT and ETL essential oils, EQC, MER and TOB was determined using the microdilution method in a 96‐well microplate according to the Clinical and Laboratory Standards Institute (CLSI 2025). Briefly, in each well 95 μL of Mueller‐Hinton (MH) broth (Oxoid Ltd., Basingstoke, UK) and 5 μL of bacterial suspensions were added for a final inoculum concentration of 10^5^ colony‐forming unit (CFU)/mL. Then, 100 μL of EOs, EQC, MER, and TOB serial dilutions were added to obtain concentrations ranging from 1024 to 0.125 µg/mL.

The negative control wells consisted of bacteria in MH without antibiotics and without EOs or EQC. The plates were mixed on a plate shaker at 300 rpm for 30 s and incubated at 37°C for 24 h. To ensure an accurate quantitative assessment and account for the baseline turbidity of the essential oils, MICs were determined using a microtiter plate reader (iMark, Bio‐Rad, CA, USA) at 595 nm. The recorded OD values were normalized by subtracting the absorbance of background controls (blanks) prepared for each specific concentration of EOT, ETL, and EQC in sterile MH broth without inoculum. Following this normalization, the MIC was defined as the lowest concentration that inhibited growth, evidenced by a final OD_595_ value ≤ 0.05. The minimum bactericidal concentration (MBC) values of EOT, ETL EQC, and the antibiotics MER and TOB were determined by sub‐culturing 5–10 μL with concentrations equal or higher than MIC on MH agar. The MBC was defined as the lowest concentration of the EOs, EQC, and the antibiotics tested, required to kill 99.99% of the bacteria. All the experiments were conducted in triplicate and results were represented by the arithmetic mean of the three values.

### 2.4 Checkerboard Dilution

The interaction of MER and TOB in combination with EOT, ETL, and EQC was used to evaluate their synergistic effect using the broth microdilution checkerboard method as previously described (Bellio et al. 2021). Briefly, a two‐fold serial dilution was used to achieve a final concentration range of 0.5–512 µg/mL for estragole (EOT, ETL, and EQC), and 0.015–128 µg/mL for MER and TOB in 96‐well microtiter plates. The MIC of each agent alone was determined on the same plate as the combinations to ensure an accurate and internally consistent calculation of the FICI, accounting for any potential inter‐assay variability. Then, a 100 µL inoculum, equal to 5 × 10^5^ CFU/mL from bacteria, was distributed into each well and incubated for 24 h at 37°C.

For the analysis of the combined antimicrobial effect, the Fractional Inhibitory Concentration Index (FICI) was used, based on the MIC values of the combined compounds divided by the MIC value of the individual component. The FICI was calculated using the following formula:

FICI=FICIa+FICIb

$$\text{FICIa}=\frac{\text{MIC of each EOs or EQC in combination with MER or TOB}}{\text{MIC}\;\text{of each EOs or EQC alone}}$$

$$\text{FICIb}=\frac{\text{MIC of MER or TOB in combination with EOs or EQC}}{\text{MIC of MER or TOB alone}}$$

The FICI values were interpreted as synergistic if FICI ≤ 0.5, as additive if 0.5 < FICI ≤ 1, indifferent if 1 < FICI ≤ 4.0, and antagonistic if > 4 (Feng and Yang 2023).

The MIC gain factor of meropenem and tobramycin was calculated according to the following formula:

$$\text{MICGF}=\frac{\text{MIC of MER or TOB alone}}{\text{MIC}\;\text{of MER or TOB in combination with EOs or EQC alone}}$$

## 3 Results

Table 3 shows the results of the antibacterial activity of EOT, ETL, and EQC, as well as the antibiotics MER and TOB against various MDR bacterial strains. Regardless of its origin, estragole exhibited an antimicrobial effect against all tested strains at low concentrations. For the group of control strains without resistance markers, the inhibitory range of estragole was between 4 and 32 µg/mL, with bactericidal activity occurring at concentrations between 8 and 128 µg/mL. For strains grouped within the MDR category, the inhibitory concentrations of estragole ranged from 16 to 128 µg/mL, with MBC values being at least two dilutions higher than MIC for each strain. Bacteria with an XDR phenotype were susceptible to estragole at concentrations between 32 and 256 µg/mL, with a bactericidal effect observed at concentrations between 64 and 512 µg/mL. In general, the bactericidal power of estragole was no greater than two dilutions higher than the MIC. On the other hand, the MIC results for MER and TOB antibiotics confirmed the previously recorded susceptibility profile of the study strains. All XDR strains showed resistance to MER, and of these, only strain *P. aeruginosa* LMM‐022 was also resistant to TOB. The remaining bacteria studied were sensitive to MER and TOB.

**Table 3:** MIC (µg/mL) and MBC (µg/mL) of estragole from *O. tenuiflorum* (EOT) and *T. lucida* (ETL), chemical pure estragole (EQC) and meropenem (MER) and tobramycin (TOB) against MDR and XDR Gram negative bacteria of clinical origin.

			EOT		ETL		EQC		MER		TOB	
N° strain		Bacteria	MIC	MBC	MIC	MBC	MIC	MBC	MIC	MBC	MIC	MBC
Sensible (S)^a^												
ATCC 25922	*E. coli*		8	16	8	16	4	8	0.06	0.125	0.125	0.25
ATCC 27853	*P. aeruginosa*		32	128	32	64	16	32	0.5	0.5	0.25	0.25
Multi‐drug resistant (MDR)												
LMM‐19B2	*E. coli*		64	128	32	64	64	256	1	2	2	2
LMM‐U337	*E. cloacae*		64	256	64	256	16	64	0.5	2	1	2
LMM‐2009	*K. pneumoniae*		64	128	128	512	128	256	1	2	1	4
LMM‐029	*K. pneumoniae*		16	64	16	64	64	128	2	4	2	4
LMM‐179	*S*. Heidelberg		32	128	64	128	128	512	0.5	1	0.50	1
Extensively drug‐resistant (XDR)												
LMM‐A717	*E. coli*		64	128	64	128	128	512	16	NA	2	4
LMM‐U2023	*K. pneumoniae*		32	64	32	128	32	128	16	NA	4	16
LMM‐U496	*A. baumannii*		256	512	128	256	128	256	64	NA	4	8
LMM‐022	*P. aeruginosa*		128	256	128	512	128	512	64	NA	16	NA
LMM‐14249/2	*P. alcaligenes*		32	64	64	512	32	64	32	NA	1	2

Abbreviations: MIC, minimum inhibitory concentration; MBC, minimum bactericidal concentration.

a Bacterial strains used for quality control of antimicrobial susceptibility tests.

The results of the antibacterial activity of EOT, ETL, and EQC in combination with the selected antibiotics (MER and TOB) are shown in Table 4. The antibacterial activity of estragole in combination with MER and TOB resulted in a substantial decrease in the MIC for all the strains studied. Interaction between estragole, from any of the sources studied here, and MER or TOB, showed a significant reduction in MIC, with inhibitory effects ranging from 0.015 to 64 µg/mL in any of their combination. As shown in Table 5, the evaluation of the association between estragole and MER or TOB exhibited synergistic activity in over 88.9% of the analyzed strains, while an additive effect or partial synergism was observed in the remaining 11.1% of strains. The synergistic activity between estragole and MER was evident in strains with phenotypic sensibility, with FICI values ranging from 0.26 to 0.50. In contrast, the FICI for the combination of estragole and tobramycin ranged from 0.14 to 0.50. The lowest FICI was found in the MDR bacteria group for the combination ETL + TOB in strains *E. cloacae* LMM‐U337 (FICI = 0.24) and *S*. Heidelberg LMM‐179 (FICI = 0.27), and for the combination EQC + TOB in strain *E. coli* LMM‐A717 (FICI = 0.31). An additive effect was observed in specific instances among the XDR strain group. Notably, the ETL + MER and ETL + TOB combinations demonstrated an additive interaction (FICI: 0.75) in one of the five tested strains (20%), specifically the *P. aeruginosa* LMM‐022 strain. All the combinations of estragole and selected antibiotics reduced the MIC values. The gain factor, which expresses the reduction in the MIC of the MER and TOB in combination with EOT, ETL and EQC, ranged from 2‐ to 16‐fold against all the strains studied.

**Table 4:** Antibacterial activity of estragole from *O. tenuiflorum* (EOT) and *T. lucida* (ETL), chemical pure estragole (EQC) combined with meropenem (MER) and tobramycin (TOB) against MDR and XDR Gram negative bacteria of clinical origin.

N°		MIC (µg/mL)					
Strain	Bacteria	EOT + MER	EOT + TOB	ETL + MER	ETL + TOB	EQC + MER	EQC + TOB
Sensible (S)^a^							
ATCC 25922	*E. coli*	0.5/0.0125	0.125/0.0625	0.5/0.015	0.5/0.0125	0.5/0.015	0.25/0.0312
ATCC 27853	*P. aeruginosa*	8/0.125	8/0.0625	8/0.125	4/0.0625	4/0.125	4/0.0625
Multi‐drug resistant (MDR)							
LMM‐19B2	*E. coli*	16/0.250	4/0.50	4/0.25	8/0.50	16/0.250	8/0.5
LMM‐U337	*E. cloacae*	8/0.125	4/0.25	8/0.125	8/0.125	4/0.125	4/0.125
LMM‐2009	*K. pneumoniae*	16/0.50	8/0.25	32/0.25	32/0.125	32/0.25	32/0.125
LMM‐029	*K. pneumoniae*	4/1	4/0.5	4/0.5	4/0.50	8/0.50	8/0.5
LMM‐179	*S*. Heidelberg	8/0.125	8/0.125	16/0.125	16/0.125	8/0.125	8/0.125
Extensively drug‐resistant (XDR)							
LMM‐A717	*E. coli*	16/4	8/0.5	32/2	32/0.50	16/2	8/0.5
LMM‐U2023	*K. pneumoniae*	8/4	4/1	8/4	8/1	8/4	8/1
LMM‐U496	*A. baumannii*	64/8	32/2	32/16	64/1	32/4	32/1
LMM‐022	*P. aeruginosa*	32/8	32/4	64/16	64/4	16/16	32/4
LMM‐14249/2	*P. alcaligenes*	8/8	16/0.50	16/8	16/0.250	4/8	16/0.0125

a Bacterial strains used for quality control of antimicrobial susceptibility tests.

**Table 5:** Evaluation of the antibacterial interaction of estragole from *O. tenuiflorum* (EOT) and *T. lucida* (ETL), chemical pure estragole (EQC) combined with meropenem (MER) and tobramycin (TOB) against MDR and XDR Gram negative bacteria of clinical origin.

		EOT associated with						ETL associated with						EQC associated with					
Sensible		MER			TOB			MER			TOB			MER			TOB		
(S)*		FICI	Int	Gain	FICI	Int	Gain	FICI	Int	Gain	FICI	Int	Gain	FICI	Int	Gain	FICI	Int	Gain
ATCC 25922	*E. coli*	0.30	S	3	0.50	S	4	0.26	S	6	0.14	S	4	0.32	S	6	0.30	S	4
ATCC 27853	*P. aeruginosa*	0.50	S	4	0.49	S	4	0.50	S	4	0.37	S	4	0.50	S	4	0.49	S	4
Multi‐drug resistant (MDR)																			
LMM‐19B2	*E. coli*	0.50	S	4	0.31	S	4	0.38	S	4	0.50	S	4	0.50	S	4	0.38	S	4
LMM‐U337	*E. cloacae*	0.38	S	4	0.31	S	4	0.38	S	4	0.24	S	8	0.49	S	4	0.38	S	8
LMM‐2009	*K. pneumoniae*	0.75	A	2	0.38	S	4	0.50	S	4	0.37	S	8	0.50	S	4	0.37	S	8
LMM‐029	*K. pneumoniae*	0.75	A	2	0.50	S	4	0.50	S	4	0.50	S	4	0.37	S	4	0.38	S	4
LMM‐179	*S*. Heidelberg	0.50	S	4	0.50	S	4	0.64	A	2	0.27	S	4	0.32	S	4	0.31	S	4
Extensively drug‐resistant (XDR)																			
LMM‐A717	*E. coli*	0.50	S	4	0.38	S	4	0.62	A	2	0.32	S	16	0.25	S	8	0.31	S	4
LMM‐U2023	*K. pneumoniae*	0.50	S	4	0.38	S	4	0.50	S	4	0.50	S	4	0.50	S	4	0.50	S	4
LMM‐U496	*A. baumannii*	0.38	S	8	0.65	A	2	050	S	4	0.50	S	4	0.31	S	16	0.50	S	4
LMM‐022	*P. aeruginosa*	0.38	S	8	0.50	S	4	0.75	A	2	0.75	A	2	0.38	S	4	0.50	S	4
LMM‐14249/2	*P. alcaligenes*	0.50	S	4	1	A	2	0.50	S	4	0.50	S	4	0.38	S	4	0.50	S	8

Abbreviations: A, addition; FICI, fractional inhibitory concentration index; Int, interpretation; S, synergism.

Table 6 shows the distribution of gain or reduction factor of meropenem (MER) and tobramycin (TOB) in the presence of estragole (EOT, ETL, and EQC). Results indicate that different estragole and antibiotic mixtures enhance the inhibitory effect of MER and TOB. The most frequent outcome was a reduction in the inhibitory effect of up to fourfold, corresponding to 66.7% of strains subjected to estragole and MER and 70.8% of strains inhibited by estragole and TOB. The highest MIC reductions (8‐ and 16‐fold) were observed with the estragole and TOB combinations (6 strains), while the lowest reductions (2‐fold) were found with the estragole and MER mixtures in 5 strains.

**Table 6:** Distribution of the MIC gain (reduction factor) of meropenem (MER) and tobramycin (TOB) in the presence of estragole from *O. tenuiflorum* (EOT) and *T. lucida* (ETL), and chemical pure estragole (EQC).

Reduction factor	EOT, ETL and EQC combined with					
	Meropenem		Tobramycin		Total	
Gain	No.	%	No.	%	No.	%
2	5	13.89	3	8.33	8	11.11
3	1	2.78	0	0.00	1	1.39
4	24	66.67	27	75.00	51	70.83
6	2	5.55	0	0.00	2	2.78
8	3	8.33	5	13.89	8	11.11
16	1	2.78	1	2.78	2	2.78
Total	36	100.00	36	100.00	72	100

## 4 Discussion

Antibiotic resistance is growing at an alarming rate worldwide, and stagnation in antimicrobial pharmacology innovation is exacerbating this issue (Salam et al. 2023; Karnwal et al. 2025). Consequently, therapeutic options for treating and controlling infectious diseases, particularly those caused by MDR bacteria, are becoming increasingly limited (WHO 2024). Developing innovative alternatives to classical antibiotics or discovering adjuvant substances are promising strategies for addressing this challenge. Previous studies have shown that EOs are a source of substances or active principles with antimicrobial properties (Cheesman et al. 2017; Chouhan et al. 2017). Combination therapies involving EOs with conventional antimicrobials can increase their efficacy, giving a valid option for the clinical management and control of infections caused by MDR pathogens (Boren et al. 2020).

The results obtained in this study provide solid evidence on the antimicrobial activity of estragole, the main component of the essential oils of *O. tenuiflorum* and *T. lucida*. EOT and ETL demonstrated bactericidal activity with low MICs against MDR and XDR strains, confirming their potential as an antimicrobial agent. However, there is no consensus on what constitutes an acceptable level of inhibition when comparing phytocompounds with antibiotic standards (Silva et al. 2019). Some authors argue that natural products are only effective if their inhibition values are similar to those of antibiotics for clinical use (Alam et al. 2022). By comparing the inhibitory effects of EQC with those of selected antibiotics, such as MER and TOB, the added value of estragole as a bactericidal agent in highly resistant bacterial strains, with MICs ranging from 4 to 256 µg/mL, was evident. This antimicrobial effect supports the hypothesis that estragole, as a phenylpropanoid, acts by altering cell membrane permeability and inducing oxidative stress (Yang et al. 2021; Lim et al. 2022; de Sousa et al. 2023). These mechanisms are unaffected by the AMR strategies present in the MDR and XDR strains analyzed in this study. Nevertheless, in some XDR strains, such as *A. baumannii* LMM‐U496 (MIC: 256 µg/mL) and *P. aeruginosa* LMM‐022 (MIC: 128 µg/mL), the highest MIC values for estragole were observed. This MIC variability may be attributed to the complexity of their cell membranes and intrinsic defense mechanisms, as documented by Atta et al. (2023) and SeyedAlinaghi et al. (2025).

Associations of estragole with MER and TOB revealed a significant reduction in the MIC of both antibiotics evidenced by FICI values, which were within the interpretive categories of synergism (≤ 0.5) or addition (≥ 0.5–1). Of the combinations evaluated, more than 88% exhibited synergy, while approximately 11% showed an additive effect or partial synergism. High MIC reductions of up to 16‐fold were observed in the majority of estragole associations with MER or TOB. These results were independent of the AMR profile of the strains studied. It is noteworthy that no indifference nor antagonistic effects were recorded for any of the combinations of EOT, ETL, or EQC with the selected antibiotics (MER or TOB). These findings are consistent with previous reports of the synergistic interactions between phytochemicals and conventional antibiotics against MDR pathogens (Araújo Silva et al. 2016; Gan et al. 2023). This synergistic activity clearly demonstrates that natural compounds can boost the effectiveness of antimicrobials and reverse extensive or complex resistance in difficult‐to‐treat bacteria (Suganya et al. 2022; Guedes et al. 2024).

The rising prevalence of XDR Gram‐negative bacteria, such as *A. baumannii*, *P. aeruginosa*, and Enterobacterales, poses a relevant threat to clinical control and management due to the limited therapeutic options available (WHO 2024). According to WHO recommendations, exploring innovative strategies is urgently needed to combat AMR, including the use of non‐traditional biological compounds like estragole as adjuvants or alternatives to conventional antibiotics (WHO 2022; Salam et al. 2023; WHO 2024). In this study, especially the combination of EOT or ETL with TOB exhibited a strong synergy against Enterobacterales such as *E. cloacae* LMM‐U337, *S*. Heidelberg LMM‐179, and *E. coli* LMM‐A717. This suggests possible differences in the molecular interaction of estragole with the various antibiotic mechanisms of action (Yang et al. 2021; Attar 2025). Furthermore, the additive effects observed in specific XDR strains, such as *A. baumannii* LMM‐U496 and *P. aeruginosa* LMM‐022, suggest that although synergy does not occur systematically in all bacterial groups, it is partially present in some cases, enhancing the antibacterial activity of associated antibiotics (Alam et al. 2022; Suganya et al. 2022; Mihai et al. 2025). However, synergism between estragole and reference antibiotics could be beneficial. The reduction of active antibiotic levels allows lower doses, ameliorating adverse effects and delaying the emergence of AMR (Cheesman et al. 2017; Chouhan et al. 2017; Boren et al. 2020; Rajčević et al. 2022). This is of particular importance given the limited number of new antibiotics and the urgent requirement to prolong the useful life span of current drugs (Salam et al. 2023; Mancuso et al. 2021; WHO 2022). These results highlight the importance of expanding pharmacological studies to optimize combinations according to the microorganism and its mechanisms of action (Torres‐Martínez et al. 2022; Kashyap 2024).

Concerning the toxicological profile of the compounds analyzed, it is important to highlight that although no tests were conducted to assess cytotoxicity, several studies support the safety of this phytochemical at the concentrations used in this study (Mahendra et al. 2023; Matei and Visan 2025). Estragole is classified as Generally Recognized as Safe (GRAS) by the United States Food and Drug Administration (FDA) (National Center for Biotechnology Information 2026), and research indicates that significant cellular damage is typically limited to high doses, ≥ 2000 µM ( ~ 296 µg/mL) threshold reported to trigger apoptosis (Sreepian et al. 2022; Mahendra et al. 2023; de Sousa et al. 2023; Kashyap 2024). Comparing our findings with these benchmarks reveals a favorable selectivity index (SI), particularly in synergistic combinations where estragole concentrations were reduced up to 16‐fold. This suggests that the effective antimicrobial doses achieved through synergy are substantially lower than the known toxicological limits for host cells.

Based on the results obtained, in vivo studies, toxicological evaluations, and clinical trials are essential to validate the safety and efficacy of treatments combining estragole with antibiotics in clinical settings (Guedes et al. 2024; SeyedAlinaghi et al. 2025). Incorporating estragole into therapeutic protocols could effectively enhance the action of antimicrobials against MDR and XDR bacteria, establishing a significant advancement in combating infections caused by these pathogens (Suganya et al. 2022).

## 5 Conclusion

The present study reveals the synergistic effect of EOT, ETL, and EQC associated with MER and TOB against Gram‐negative MDR and XDR bacteria. The observed synergistic interactions remarkably reduce MICs of these important antibiotics, suggesting a promising therapeutic strategy to prolong the clinical lifespan of existing antibiotics, reduce the required doses, minimize toxicity, and delay resistance.

These findings emphasize the role of phytocompounds like estragole as a valuable source of novel antimicrobial agents, a hopeful approach to combat AMR, particularly for infections caused by critical priority pathogens. However, further research is needed to fully elucidate the pharmacological aspects, bioavailability, and mechanism of interaction of such compounds in order to fully translate these findings into the development of clinically effective therapies.

This study confirms the effectiveness of natural bioactive compounds to potentiate conventional antibiotics, offering a promising alternative to confront the growing global challenge posed by AMR.

## Author Contributions

**María Araque:** conceptual design and experimental management, analysis of data, literature review, writing, review, and editing of the article. **Luis B. Rojas:** provided experimental materials, supervised the study, and conducted key analysis.

## Ethics Statement

The authors have nothing to report.

## Conflicts of Interest

The authors declare no conflicts of interest.

## Data Availability

The authors confirm that the data supporting the findings of this study are available within the article.
